# Seminuclear Flip Phacoemulsification for Soft Nuclear Cataract Surgery: A New Soft Nucleus Cataract Chopping Technique

**DOI:** 10.1155/joph/9448941

**Published:** 2026-07-31

**Authors:** Han Wang, Zebin Li, Rubing Liu, Furong Luo, Jifa Kuang, Jing Yang, Beiling Tan, Hong He, Mingbing Zeng

**Affiliations:** ^1^ State Key Laboratory of Ophthalmology, Zhongshan Ophthalmic Center, Guangdong Provincial Key Laboratory of Ophthalmology and Visual Science, Sun Yat-Sen University, 7 Jinsui Road Tianhe, Guangzhou 510623, China, sysu.edu.cn; ^2^ Hainan Eye Hospital and Key Laboratory of Ophthalmology, Zhongshan Ophthalmic Center, Sun Yat-Sen University, Haikou 570311, Hainan, China, sysu.edu.cn; ^3^ Shenzhen Eye Hospital, Jinan University, 18 Zetian Road, Shenzhen 518040, China, jnu.edu.cn

**Keywords:** cataract, phacoemulsification, quick chop, seminuclear flip

## Abstract

**Purpose:**

To introduce and evaluate the seminuclear flip phacoemulsification (SNFP), a new soft nucleus cataract phacoemulsification surgical technique, in comparison with the conventional quick‐chop phacoemulsification (QCP).

**Design:**

Prospective observational study.

**Methods:**

According to the Emery–Little Classification System for grading nuclear opacity, 176 cases with nuclear hardness of Grades 1 and 2 were divided into two groups; one group received SNFP, and the control group underwent QCP. The intraoperative ultrasound time, ultrasound energy, complications, postoperative best‐corrected visual acuity, central corneal thickness, and central corneal endothelial cell density were evaluated to determine the advantages of the new technique over conventional surgery.

**Results:**

The ultrasound time was significantly shorter in the SNFP group than in the QCP group (*p* = 0.01). The difference in the ultrasound energy between the groups was not statistically significant. Two cases of posterior capsule rupture were observed in the QCP group, while no such case was observed in the SNFP group. There was no significant difference in visual acuity or central corneal thickness between the two groups before the operation or 1 day, 7 days, or 1 month after the operation. There was no significant difference in the density of central corneal endothelial cells between the two groups before and 1 month after the operation.

**Conclusions:**

SNFP can improve the efficiency of phacoemulsification and reduce the complication risk in soft nucleus cataract surgery.

**Trial Registration:** Chinese Registry of Clinical Trials: ChiCTR2200063495

## 1. Introduction

Cataract extraction has evolved considerably from intracapsular to extracapsular methods and now phacoemulsification. After decades of development, phacoemulsification has become the main surgical method for treating cataracts owing to its high efficiency and speed while imparting minimal trauma and a short recovery time [1–3]. However, the damage to the corneal endothelium caused by phacoemulsification remains one of the main surgical complications. Especially for hard nuclear cataracts, the risk of damage to corneal endothelial cells increases with increased nuclear hardness [4, 5]. Surgical techniques for treating nuclear blocks have also evolved from the original nonsplitting technology to the current splitting technology, reducing ultrasonic energy damage during the operation [6]. Nuclear splitting has emerged as a prerequisite for the effective phacoemulsification of hard nuclear cataracts. Following the invention of the first cataract nucleus cracking technique, the divide and conquer technique [7], a variety of nuclear chopping methods have been developed, such as the phaco chop, quick chop, horizontal chop, and vertical nuclear chopping [8–10]. These cracking techniques greatly improve the efficiency of phacoemulsification and reduce the occurrence of serious complications, such as corneal injury and even corneal endothelial dysfunction [11]. The chopping methods are mainly suitable for moderate or hard nuclei but relatively unsuitable for soft nucleus cataract phacoemulsification, as the nucleus is too soft to be aspirated and grasped in the phaco tip; thus, subsequent splitting is challenging. Without nucleus splitting, the surface of the soft nucleus easily forms a bowl‐shaped nuclear residue, thereby resulting in a greater challenge for subsequent phacoemulsification and greatly increasing the risk of posterior capsule rupture. Thus, exploring an efficient technique for soft nucleus cataract surgery is crucial. Dr. Om Parkash introduced the RAPID method for soft nuclei, an en masse nonfragmentation technique solely for soft cataracts [12]. However, it can be difficult to distinguish whether specific cases are purely soft nuclei. Rotation and axis‐rotation techniques for soft cataract surgery have also been reported [13, 14]; however, rotating certain soft nuclei can be challenging, requiring the surgeon to resort to the splitting technique.

Against the drawbacks of conventional chopping and existing soft‐nucleus cataract procedures, including difficult nuclear grasping, unavoidable residual bowl‐shaped cortex, and elevated risk of posterior capsule tear documented in previous research studies, we developed an innovative seminuclear flip phacoemulsification (SNFP) technique. This newly designed approach is hypothesized to shorten phacoemulsification duration, boost surgical efficiency, and cut down intraoperative complication rates for Grades 1–2 soft nuclear cataracts. Accordingly, the present study was carried out to detail the standardized operative steps of SNFP and systematically clarify its strengths and limitations by comparing surgical outcomes with conventional quick‐chop phacoemulsification (QCP) [15, 16].

## 2. Methods

### 2.1. Study Participants

This prospective study was conducted at Hainan Eye Hospital from September 2022 to December 2022 and registered with the China Clinical Research Registry. The study was conducted in accordance with the tenets of the Declaration of Helsinki and approved by the Ethics Committee of Hainan Eye Hospital. Written informed consent was obtained from the patients after ensuring that their personal and private information would be protected.

### 2.2. Inclusion and Exclusion Criteria

Age‐related cataracts were diagnosed if the patient was older than 50 years old. Patients with Grades 1 and 2 cataracts according to the Emery–Little classification of nuclear opacity grade were included in this study. Other inclusion criteria included the patient’s ability to return for follow‐up visits, preoperative central corneal endothelial cell density count > 1200/mm^2^, and preoperative pupil dilation > 7 mm. The exclusion criteria included a history of internal ocular surgery and other eye diseases affecting eyesight, such as corneal diseases affecting visual acuity, diabetes retinopathy, progressive glaucoma, retinitis pigmentosa, age‐related macular degeneration, uveitis, and optic neuropathy. Patients with an ocular axis length < 22 mm and > 28 mm, a fragile lens suspensory ligament, or lens subluxation were also excluded from this study.

### 2.3. Randomized Design and Sample Size Estimation

#### 2.3.1. Sample Size Estimation

Based on the principles of randomized block design, the sample size was calculated via statistical software using the mean difference in ultrasound surgical time (UST) between the experimental and control groups derived from our preliminary pilot data. A total of 88 patients in each group were enrolled to ensure adequate statistical power for detecting a clinically meaningful intergroup difference between the SNFP and QCP groups, with an allowance for an anticipated 15% loss to follow‐up.

#### 2.3.2. Randomization Method

A randomized block design stratified by lens nuclear hardness (Grade I vs. Grade II) was used. Within each stratum, an independent allocation sequence was generated using a block size of 4, with each block containing two assignments to each surgical group in random order (e.g., A, B, B, and A). Sequentially numbered, sealed, opaque envelopes were prepared for each stratum. After confirming eligibility and nuclear grade, a research coordinator not involved in outcome assessment opened the next envelope for that stratum and assigned the patient accordingly, ensuring allocation concealment.

### 2.4. Routine Preoperative Examinations and Intraocular Lens (IOL) Power Calculation

All patients underwent standard preoperative examinations, including anterior segment analysis, central corneal endothelium cell counting, Type A and Type B ultrasound, laser fundus retinography, and IOL measurement and calculation. A Pentacam AXL (Pentacam, Münchholzhäuser, Germany) was used to measure corneal astigmatism. Central corneal endothelial cell density was measured using a Topcon Sp‐2000 endothelial corneal cell counter (Topcon, Tokyo, Japan). The central corneal thickness was measured by AS‐OCT (Carl Zeiss Meditec, Jena, Germany), and IOL measurements were obtained with a Zeiss IOLMaster 700 (Carl Zeiss Meditec, Jena, Germany) and the Barrett II IOL calculation formula.

## 3. Surgical Procedures

All operations were performed by author Mingbing Zeng, who has extensive experience in cataract phacoemulsification and IOL implantation. An Infiniti phacoemulsification cataract surgery treatment system was used for performing the operation (Alcon, California, USA); 100% torsional continuous linear energy release mode was selected, and the vacuum and flow were set to 350 mmHg and 35 mL/min, respectively.

Standard procedures included making a 2.8‐mm temporal transparent corneal incision, injecting viscoelastic to stabilize the anterior chamber, and continuous central capsulorhexis of 5.0–5.5 mm. Each patient received either the SNFP or conventional QCP in accordance with random allocation. Lens cortex extraction and IOL implantation were routinely performed in both groups.

**FIGURE 1:**
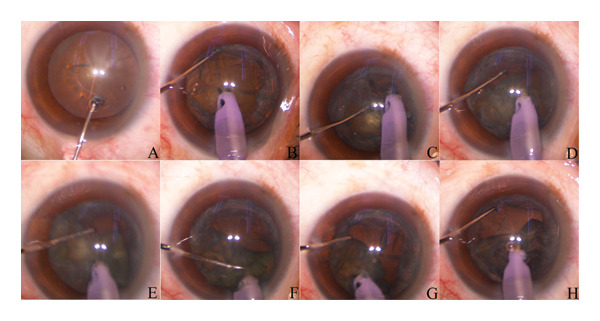
Operational steps of the seminuclear flip phacoemulsification technique (A) Preparation of the soft nuclear cataract for surgery. (B) The phacoemulsification tip enters the center of the nucleus, and the splitting hook is placed at the equator of the nucleus to prepare for splitting. (C) The phacoemulsification tip is maintained in the center of the nucleus, and the chopping hook completes the first chop from the periphery to the center. (D) The split half of the nucleus is flipped from bottom to top with the splitting hook. (E) The upturned nucleus is aspirated and dragged into the anterior chamber. (F) The first half of the nucleus is emulsified in the anterior chamber. (G) The other half of the nucleus is aspirated by the phacoemulsification tip and pulled into the anterior chamber. (H) The other half of the nucleus is emulsified in the center of the anterior chamber.

**FIGURE 2:**
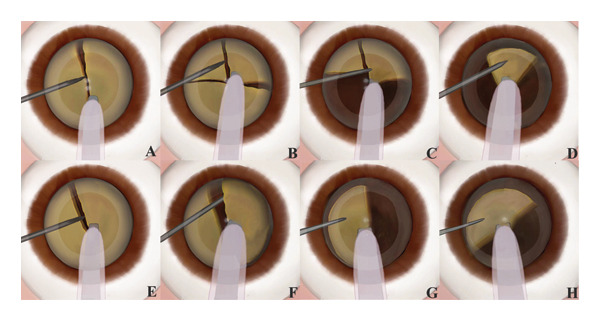
Schematic diagram comparing the quick‐chop phacoemulsification (QCP) and seminuclear flip phacoemulsification (SNFP) operation steps. Figures A to D show the conventional QCP technique. (A) The nucleus is cracked into two pieces. (B) Through a second splitting, half of the nucleus is split into two‐quarter pieces to be emulsified. (C) After half of the nucleus is emulsified, the remaining half is split into two pieces. (D) The last nuclear mass is removed by phacoemulsification. Figures E to H show the SNFP technique. (E) The phacoemulsification tip is embedded in the center of the nucleus, and the nucleus is split by the cracking hook. (F) The cracking hook is placed under the cleaved hemisected nucleus to flip it from the bottom to the top. The phacoemulsification tip is then used to aspirate the raised half of the nucleus, and emulsification and aspiration are performed in the anterior chamber. (G) The phacoemulsification tip is used to aspirate the other half of the nucleus. (H) The last half of the nucleus is aspirated and pulled into the anterior chamber for emulsification. SNFP: seminuclear flip phacoemulsification; QCP: quick‐chop phacoemulsification.

SNFP technique: The SNFP technique begins by inserting the phacoemulsification tip until it reaches the center of the nucleus. Then, the splitting hook is used to bisect the nucleus from the equator to the center (Figures 1A–C and 2E), and half of the nucleus on the opposite side of the nuclear splitting hook is flipped up from the bottom to the top, and then subsequently aspirated and emulsified in the anterior chamber (Figures 1D–F and 2F). The phacoemulsification tip is then used to suction the other half of the nucleus and drag it into the anterior chamber for emulsification (Figures 1G and H and 2G) (Video S2).

QCP technique: The QCP was described previously [15, 16]. In brief, after the phacoemulsification tip enters the center of the nucleus, as much of the nucleus as possible is removed, and then the nuclear splitting hook is used to split the nuclear block from the equator to the center (Figure 2A). The nuclear block is next rotated and split for a second time; the split nuclear block is gently removed and emulsified either in situ or in the anterior chamber (Figure 2B). The other half of the nucleus is rotated and split (Figure 2C) and also emulsified either in situ or in the anterior chamber (Figure 2D) (Video S1).

After the operation, tobramycin/dexamethasone eye suspension 0.3%/0.05% (Tobradex, Alcon, Puurs, Belgium) and antibiotics (0.5% levofloxacin, Santen, Osaka, Japan) were administered locally four times a day, and the dose was gradually decreased after two weeks.

### 3.1. Intraoperative and Postoperative Observation Indicators

The intraoperative ultrasound energy (cumulative dissipated energy [CDE]), ultrasound time (UST), and complications were recorded. Postoperative follow‐up and examination were performed by investigators blinded to the operation assignment. The best‐corrected visual acuity, central corneal thickness, and corneal endothelial cell count before and 1 month after the operation were examined and recorded.

### 3.2. Statistical Methods

All statistical analyses were conducted using IBM SPSS Statistics 26.0 (IBM Corporation, Armonk, NY, USA). Depending on the type and distribution of collected data, independent samples *t*‐test, one‐way analysis of variance (ANOVA), and chi‐square test were adopted for intergroup comparisons. A *p* value < 0.05 was defined as statistically significant.

## 4. Results

### 4.1. Demographics and Hardness Grading of the Cataract Nuclei

A total of 87 and 89 patients were included in the SNFP and QCP groups, respectively. The average ages of the patients in the two groups were 57.7 ± 4.3 and 56.9 ± 4.3 years, respectively; the difference between the two groups was not statistically significant by the *t*‐test (*p* = 0.24). 30 and 57 cataracts with hardness grades of 1 and 2 were present in the SNFP group, respectively, while 29 and 60 cataracts were present in the QCP group, respectively. The difference in the distribution between the two groups was not statistically significant by the chi‐square test (*p* = 0.78) (Table 1).

**TABLE 1:** Comparison of baseline data between the two groups of cataract patients.

Observation index	SNFP group (*n* = 87)	QCP group (*n* = 89)	Statistical value	*p* value
Mean age (years)	57.7 ± 4.3	56.9 ± 4.3	*t* = 1.18	0.24

Nuclear hardness grade 1	30 cases	29 cases	*χ* ^2^ = 0.08	0.78
Nuclear hardness grade 2	57 cases	60 cases		

*Note:* SNFP, seminuclear flip phacoemulsification.

Abbreviation: QCP, quick‐chop phacoemulsification.

### 4.2. Comparison of Intraoperative Parameters and Complications

**FIGURE 3:**
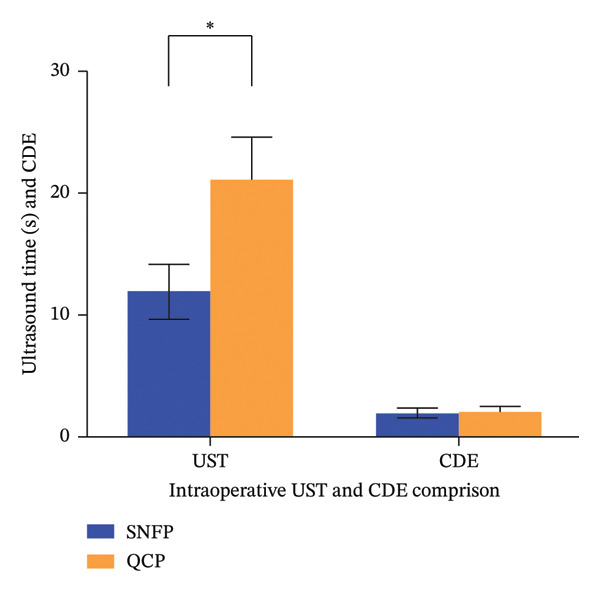
Comparison of intraoperative ultrasound time and cumulative dissipated energy. Comparison of the intraoperative ultrasound times showed that the SNFP group required significantly less ultrasound time than the QCP group. The SNFP group also needed less ultrasound energy than the QCP group; however, the difference was not statistically significant. CDE: cumulative dissipated energy; UST: ultrasound time; SNFP: seminuclear flip phacoemulsification; QCP: quick‐chop phacoemulsification. ^∗^Statistically significant (*p* < 0.05).

The average USTs in the SNFP and QCP groups were 11.91 ± 2.24 s and 21.05 ± 3.51 s, respectively. The average UST in the SNFP group was significantly lower than that in the QCP group (*t*‐test, *p* = 0.01) (Figure 3). The average CDE was 1.97 ± 0.42 and 2.04 ± 0.47 in the SNFP and QCP groups, respectively; the difference between the SNFP and QCP groups was not statistically significant (*t*‐test, *p* = 0.26) (Figure 3). Two cases of posterior capsule rupture occurred in the QCP group; however, none occurred in the SNFP group, and the difference between the two groups was statistically significant (Fisher exact test, *p* = 0.02). For the two patients in the control group who experienced posterior capsule rupture, anterior vitrectomy and ciliary sulcus IOL implantation were performed. Only their complication data were included in intergroup comparisons, while all other intraoperative and postoperative outcome data from these two cases were excluded (Table 2).

**TABLE 2:** Comparison of intraoperative parameters and complications.

Observation index	SNFP group (*n* = 87)	QCP group (*n* = 89)	Statistical value	*p* value
Average UST (s)	11.91 ± 2.24	21.05 ± 3.51	*t* = 2.59	0.01
Average CDE	1.97 ± 0.42	2.04 ± 0.47	*t* = 1.13	0.26
Posterior capsule rupture	0 cases	2 cases	Fisher exact	0.02

*Note:* UST, ultrasound time; SNFP, seminuclear flip phacoemulsification.

Abbreviations: CDE, cumulative dissipated energy; QCP, quick‐chop phacoemulsification.

### 4.3. Best‐Corrected Visual Acuity Comparison Before and After Operation

The average preoperative best‐corrected visual acuity was 0.57 ± 0.19 logarithm of the minimum angle of resolution (LogMAR) and 0.55 ± 0.20 LogMAR in the SNFP and QCP groups, respectively; the difference between the two groups was not statistically significant (*t*‐test, *p* = 0.66). The average visual acuity one day after the operation in the SNFP group was 0.117 ± 0.09 LogMAR and that in the QCP group was 0.120 ± 0.08 LogMAR; the difference was not statistically significant (*t*‐test, *p* = 0.64). The average best‐corrected visual acuity 7 days and 1 month after the operation was 0.038 ± 0.086 and −0.049 ± 0.0728 LogMAR in the SNFP group and 0.044 ± 0.090 and −0.065 ± 0.068 LogMAR in the QCP group, respectively. There were no significant differences between the two groups (*t*‐test, *p* = 0.2 and 0.12, respectively) (Table 3).

**TABLE 3:** Best‐corrected visual acuity comparison before and after operation.

Observation index	SNFP group (*n* = 87)	QCP group (*n* = 89)	Statistical method	*p* value
Preoperative average BCVA (LogMAR)	0.57 ± 0.19	0.55 ± 0.20	*t* = 0.44	0.66
1 day postoperative average BCVA (LogMAR)	0.117 ± 0.09	0.120 ± 0.08	*t* = 0.47	0.64
7 days postoperative average BCVA (LogMAR)	0.038 ± 0.086	0.044 ± 0.090	*t* = 1.29	0.2
1 month postoperative average BCVA (LogMAR)	−0.049 ± 0.073	−0.065 ± 0.068	*t* = 1.56	0.12

*Note:* SNFP, seminuclear flip phacoemulsification.

Abbreviations: BCVA, best‐corrected visual acuity; QCP, quick‐chop phacoemulsification.

### 4.4. Comparison of the Preoperative and Postoperative Central Corneal Thickness and Endothelial Cell Density

The differences in preoperative central corneal thickness and endothelial cell density between the SNFP and QCP groups were not statistically significant. The average central corneal thickness was 555.1 ± 29.1 μm and 551.3 ± 30.9 μm in the SNFP and QCP groups, respectively. No significant difference was observed between the two groups (*t*‐test, *p* = 0.35). The mean central corneal endothelial cell density was 2430.9 ± 263.5 cells/mm^2^ and 2349.1 ± 287.3 cells/mm^2^ in the SNFP and QCP groups, respectively, but the difference was not significant (*t*‐test, *p* = 0.18). The average central corneal thickness one day after the operation was 576.7 ± 46.3 μm and 581.3 ± 58.3 μm in the SNFP and QCP groups, respectively. No significant difference was observed between the two groups (*t*‐test, *p* = 0.17); similarly, no significant differences were observed in the central corneal thickness between the two groups at 7 days and 1 month after the operation. One month after the operation, the average central corneal endothelial cell density was 2308.6 ± 259.3 cells/mm^2^and 2231.9 ± 267.6 cells/mm^2^ in the SNFP and QCP groups, respectively. No significant difference was observed between the two groups (*t*‐test, *p* = 0.27). After adjusting for preoperative baseline corneal endothelial cell density via ANCOVA, there was no statistically significant difference in 1‐month postoperative central corneal endothelial cell density between the SNFP and QCP groups (adjusted mean: 2302.1 ± 259.3 vs. 2245.8 ± 267.6 cells/mm^2^; *F* = 1.58, *p* = 0.21). Preoperative baseline density showed no significant confounding effect on postoperative values (*p* = 0.79) (Table 4).

**TABLE 4:** Comparison of preoperative and postoperative central corneal thickness and endothelial cell density.

Observation index	SNFP group (*n* = 87)	QCP group (*n* = 89)	Statistical method	*p* value
Preoperative central corneal thickness (μm)	555.1 ± 29.1	551.3 ± 30.9	*t* = 0.94	0.35
1 day postoperative central corneal thickness (μm)	576.7 ± 46.3	581.3 ± 58.3	*t* = 1.38	0.17
7 days postoperative central corneal thickness (μm)	553.2 ± 28.7	550.8 ± 30.2	*t* = 0.54	0.59
1 month postoperative central corneal thickness (μm)	554.5 ± 29.3	552.1 ± 30.5	*t* = 0.52	0.60
Preoperative central corneal endothelial cell density (cells/mm^2^)	2430.9 ± 263.5	2349.1 ± 287.3	*t* = 1.97	0.18
1 month postoperative central corneal endothelial cell density (cells/mm^2^)	2308.6 ± 259.3	2231.9 ± 267.6	*t* = 1.11	0.27

*Note:* SNFP, seminuclear flip phacoemulsification.

Abbreviation: QCP, quick‐chop phacoemulsification.

## 5. Discussion

The performance of phacoemulsification cataract surgery has gradually improved in recent decades, resulting in more efficient cataract surgery and reducing the possibility of corneal endothelial injury attributed to ultrasonic energy [17]. However, more ultrasonic energy is required for treating hard nuclear cataracts; thus, the possibility of corneal endothelial injury is greatly increased. Especially in developing countries, a high proportion of patients with hard nuclear cataracts continue to await surgery [18].

The emergence of nuclear splitting technology has greatly improved the efficiency of phacoemulsification in hard nuclear cataract cases. Consequently, a variety of different nuclear splitting methods have emerged, all of which recommend suctioning the nucleus with a high vacuum, and then dividing it into several pieces by mechanical force and emulsifying it as far from the corneal endothelium as possible to reduce corneal endothelium damage.

Compared to hard nuclear cataracts, soft nuclear cataracts require less ultrasonic energy and have a lower chance of corneal endothelial injury with treatment. Moreover, soft nuclear cataract surgery has inherent characteristics that differentiate it from hard nuclear cataract surgery. For example, the nucleus is soft and difficult to remove, and rotating the nucleus in the capsule and splitting it for the second time are challenging. Therefore, the hard nuclear cataract splitting technique currently in use is essentially unsuitable for soft nuclear cataracts. In addition, since absorption is challenging, cataract surgeons have noted that it is very easy to absorb the core and leave a thin, bowl‐shaped remnant. Thus, the surgeon faces a major dilemma: if the depth of the pedal and the ultrasonic energy and vacuum are increased, absorbing the posterior capsule is possible, thereby resulting in rupture. However, if the ultrasonic energy and negative pressure are not increased, suction and grasping of the nuclear mass away from the posterior capsule become more difficult. Certain nonsplitting phacoemulsification techniques have been suggested to solve this dilemma [13, 14, 19, 20]. However, each of these methods has its own limitations and cannot be applied to most cases, thereby limiting applicability.

The greatest advantage of the SNFP technique is that after the initial bisection, no second chopping is needed, and the bisected half‐core block is flipped up without nuclear rotation. This original SNFP technique bears similarities to the vertical chop method used in dense cataract surgery, as both involve force application from the lens periphery toward the central axis. However, it requires significantly less force compared to conventional vertical chopping. Unlike the vertical chop technique, which necessitates two‐stage nuclear splitting with subsequent rotation, this method allows direct elevation of the hemisected nucleus from the inferior to the superior pole for immediate phacoemulsification.

Because the nucleus is soft and not much energy is needed, there is no need to worry about excessive damage to the corneal endothelium even if phacoemulsification is carried out in the anterior chamber. Thus, the unsuitability of soft nuclear cataracts for rotation and secondary splitting is resolved. If the first half of the core is emulsified, the second half of the core is easily aspirated and quickly removed. Our study found that compared to the QCP group, the SNFP group had an approximately 43.4% lower UST. The duration of emulsification was shortened, and the amount of perfusion fluid and time of corneal endothelial cell washing were correspondingly reduced, all of which are beneficial for reducing corneal cell damage and the occurrence of corneal edema after surgery. Compared to other soft‐nucleus techniques, SNFP offers several practical advantages. The hemi‐flip technique relies on partial nuclear rotation to expose the inferior hemisphere, which can be challenging when the nucleus is extremely soft and lacks the rigidity needed for rotation [21]. The tilt‐and‐crush approach similarly depends on controlled nucleus tilt, which may be difficult in eyes with a small pupil or a shallow anterior chamber [22]. In contrast, SNFP requires only a single chopping step followed by direct elevation of the hemisected nucleus into the anterior chamber, without the need for secondary nuclear rotation or repeated instrument insertion. This streamlined approach reduces intraocular manipulation and may contribute to the lower posterior capsule rupture rate observed in our study.

Two cases of posterior capsule rupture were observed in the QCP group. In the SNFP technique, the divided nucleus is elevated away from the posterior capsule for anterior‐chamber phacoemulsification, which greatly improves surgical safety and reduces posterior capsule rupture risk compared to in situ emulsification. Moreover, with the emergence of new technologies, the new procedure could be updated to further improve surgical efficacy.

Although a significant intergroup difference was detected in UST and a lower incidence of posterior capsule rupture was achieved in the present study, no statistically significant differences were found regarding intraoperative total ultrasound energy, postoperative central corneal thickness, best‐corrected visual acuity, and central corneal endothelial cell density. This phenomenon may be attributed to the enrolled patients with Grades 1–2 nuclear sclerosis, who required limited intraoperative ultrasonic energy during phacoemulsification, resulting in marginal intergroup gaps in total ultrasound consumption. Theoretically, prolonged UST corresponds to increased consumption of balanced salt solution (BSS). A sufficiently enlarged sample size would probably yield statistically significant between‐group differences in the above nonsignificant indicators; however, such large‐scale enrollment would substantially raise human and financial costs. Therefore, the sample size of this trial was initially calculated based on UST as the primary outcome parameter, and future prospective trials can be designed with total ultrasound energy set as the primary endpoint for sample size estimation.

Limitations of the current study are the novelty of the SNFP technique and that all conclusions were obtained from a single surgeon’s clinical practice. Replication of consistent outcomes after implementation of this technique by multiple surgeons and different surgical centers would enhance the persuasiveness of the results and promote its wider clinical application. Further large‐scale comparative studies concerning various soft nucleus chopping techniques should be carried out in follow‐up research.

The SNFP method described in this study is a modification of the original chopping method, and thus it can be effectively used only after mastering the original chopping skills. This procedure should be avoided by beginners or less skilled operators to reduce the risk of posterior capsule rupture.

In conclusion, compared to conventional chopping and nonchopping phacoemulsification methods, SNFP can greatly improve the treatment efficiency of soft nuclear cataract phacoemulsification surgery and reduce the risk of complications.

## Funding

This supported was supported in part by an unrestricted grant from the National Natural Science Foundation of China (Grant no. 82060176), the Natural Science Foundation of Hainan Province (Grant no. 822MS190), and the Hainan Province Eye Clinical Medical Center.

## Conflicts of Interest

The authors declare no conflicts of interest.

## Supporting Information

Additional supporting information can be found online in the Supporting Information section.

## Supporting information


**Supporting Information 1** Video S1: A side port incision was created with a 15‐degree knife, followed by the construction of a 2.8‐mm wide self‐sealing temporal clear corneal incision, with ProVisc instilled to maintain the anterior chamber. A continuous curvilinear capsulorhexis of 5.0–5.5 mm was fashioned using a cystotome. After successful hydrodissection and hydrodelineation, superficial cortex was aspirated. Phacoemulsification of the nucleus proceeded with a standard quick‐chop technique, wherein the nucleus was first cracked into two halves. One‐half was then subdivided into quarters for emulsification, followed by similar splitting and removal of the remaining half. Subsequent aspiration of the peripheral cortex cleared the capsular bag for the implantation of an IOL. The lens haptics were fully unfolded, and the IOL was centered within the bag. All remaining viscoelastic agent was aspirated. Finally, stromal hydration was performed to ensure a water‐tight seal of the clear corneal incision.

**Supporting Information 2** Video S2: A side port incision was created with a 15‐degree knife, followed by the construction of a 2.8‐mm wide self‐sealing temporal clear corneal incision, with ProVisc instilled to maintain the anterior chamber. A continuous curvilinear capsulorhexis of 5.0–5.5 mm was fashioned using a cystotome. After successful hydrodissection and hydrodelineation, superficial cortex was aspirated. Phacoemulsification of the nucleus proceeded with the SNFP technique, wherein the phacoemulsification tip was embedded in the center of the nucleus, and the nucleus was split by the cracking hook. The cracking hook was placed under the cleaved hemisected nucleus to flip it from the bottom to the top. The phacoemulsification tip was then used to aspirate the raised half of the nucleus, and emulsification and aspiration were performed out of the capsule. After that, the last half of the nucleus was aspirated and pulled out of the capsule for emulsification. Subsequent aspiration of the peripheral cortex cleared the capsular bag for the implantation of an IOL. The lens haptics were fully unfolded, and the IOL was centered within the bag. All remaining viscoelastic agent was aspirated. Finally, stromal hydration was performed to ensure a water‐tight seal of the clear corneal incision.

## Data Availability

The data that support the findings of this study are available from the corresponding author upon reasonable request.
